# An Updated Review on the Multifaceted Therapeutic Potential of *Calendula officinalis* L.

**DOI:** 10.3390/ph16040611

**Published:** 2023-04-18

**Authors:** Kiran Shahane, Madhuri Kshirsagar, Srushti Tambe, Divya Jain, Srutee Rout, Maria Karolina Martins Ferreira, Suraj Mali, Purnima Amin, Prem Prakash Srivastav, Jorddy Cruz, Rafael Rodrigues Lima

**Affiliations:** 1 Department of Pharmaceutical Sciences and Technology, Institute of Chemical Technology, Mumbai 400019, India; 2 Department of Agricultural and Food Engineering, Indian Institute of Technology, Kharagpur 721302, India; 3 Laboratory of Functional and Structural Biology, Institute of Biological Sciences, Federal University of Pará, Belém 66075-110, Brazil; 4 Department of Pharmaceutical Sciences & Technology, Birla Institute of Technology, Mesra 835215, India

**Keywords:** *Calendula officinalis*, traditional medicine, chemical composition, biological activities

## Abstract

*Calendula officinalis* Linn. (CO) is a popular medicinal plant from the plant kingdom’s Asteraceae family that has been used for millennia. This plant contains flavonoids, triterpenoids, glycosides, saponins, carotenoids, volatile oil, amino acids, steroids, sterols, and quinines. These chemical constituents confer multifaceted biological effects such as anti-inflammatory, anti-cancer, antihelminthic, antidiabetes, wound healing, hepatoprotective, and antioxidant activities. Additionally, it is employed in cases of certain burns and gastrointestinal, gynecological, ocular, and skin conditions. In this review, we have discussed recent research from the last five years on the therapeutic applications of CO and emphasized its myriad capabilities as a traditional medicine. We have also elucidated CO’s molecular mechanisms and recent clinical studies. Overall, this review intends to summarize, fill in the gaps in the existing research, and provide a wealth of possibilities for researchers working to validate traditional claims and advance the safe and effective use of CO in treating various ailments.

## 1. Introduction

The use of traditional medicine was found to be first implemented in Ancient Greece. According to Greek traditional knowledge, gods gave the knowledge of healing to man. Theophrastus (372–286 BC), a disciple of Aristotle, an ancient Greek philosopher, and a scientist, authored the first scientific system of plants [1]. Although not wholly aware of their exact physicochemical characteristics at the time, the human population has found additional health benefits from plants throughout history. The same components from plant sources that have a long medicinal history and are proven effective in the welfare of human health are indicated within traditional medicine. This traditional medicinal knowledge and colonial expansion through the progress of communication mediums have been transferred over the generations [1,2]. Currently, traditional medicines are becoming more popular for therapeutic use, specifically for self-treatment practices [3,4,5].

*Calendula officinalis* Linn. (CO), as an important plant within traditional medicine, has found application in the food industry [6] as well as the pharmaceutical industry [7] owing to the presence of secondary metabolites in the plant. The *Calendula* genus covers approximately 25 species, among which *C. officinalis*, *C. arvensis*, *C. tripterocarpa*, *C. stellata,* and *C. suffruticose* are the most common [8]. CO is the most studied species of *Calendula*. It has been used medicinally since the 12th century [9,10] and is known as English Marigold, Pot Marigold, Holigold, Mary Bud, Marybud, or Mary Gowles. The name *Calendula* originates from the Latin term “calends” denoting the first day of each month when the *Calendula* flower blooms. Along with this, *Calendula* has also been referred to as the “herb of the sun”, considering the efflorescence of *Calendula* flowers in the morning and their shriveling in the evening. For a long period, this traditional herb has been used to treat minor burns, wounds, and skin problems. Currently used CO medicines include pot marigold tincture and carophyllenic ointment, which both contain carotenoids derived from the flowers. It is one of the ingredients of the branded homeopathic drug Traumeel^®^, which is intended to relieve the pain and swelling brought on by sudden musculoskeletal injuries [11]. Moreover, many sources suggest using *Calendula* petal powder as an economical substitute for saffron because its coloring and flavoring aided in food products in early times [10].

CO is a self-seeding, annual plant species that grows to a height of 12–18 inches and is found near warm and humid atmospheric conditions [12]. A 5 to 7 cm composite flower head rests on the plant’s stem. The flower head consists of an epicalyx of multiple tapered lanceolate sepals, compactly overlayed on each of the two sides by glandular hairs and yellow-orange tubular florets on the interior side [9,13]. CO powder is a yellowish-brown powder with a distinctive aromatic smell and a mildly bitter taste. It contains normocytic stomata in the outer epidermis’ apical region, fragments of the corolla, covering and glandular trichomes, elongated sclerenchymatous cells, fragments of the walls of the ovaries containing brown pigment, pollen grains, fragments of stigma, and fibrous fragments. CO plants are abundantly seen in Central Europe and the Mediterranean regions [14,15]. It is also found in Middle Eastern countries, specifically Cyprus, Turkey, and Iran. In addition, *Calendula* cultivation has also been observed in India and China on a larger scale [16,17].

It is considered a safe medication when considering its therapeutic potential with a proper dose and other pharmacological indications [9,18]. Some toxicological studies have even proven the safety of acute and subacute administration of *Calendula* in terms of biochemistry and physical parameters. According to the European Medicines Agency, CO oil is classified as a herbal medical product and has a claimed LD 50 (lethal dose 50) value of 20 mL/kg of body weight [10,19]. 

This review congregates the hitherto scattered reports on the pharmacological activities of CO from the last five years. Nonetheless, we aim to highlight the importance of CO as a natural remedy for therapeutic purposes based on the positive data that has been documented in the literature. In summary, the objective of this review is to provide a summary, fill in the gaps in the existing research, and present a multitude of possibilities to researchers already working on the validation of traditional claims and the development of CO’s use in the safe and effective treatment of a variety of diseases.

## 2. Chemical Composition

Some of the important components in CO pharmacological activities belong to different classes of chemical compounds, terpenoids, flavonoids, triterpeneol esters, steroids, phenolic compounds, carotenes, triterpenoids, essential oils, quinones, fatty acids, minerals, saponins, carbohydrates, sterols, and tocopherols [20,21]. In various regions of CO, the compounds ubiquinone, tocopherol, phylloquinone, and proto-quinone were identified from quinones. From the petroleum ether extract of CO flowers, terpenoids were extracted [22,23]. Some other phytoconstituents present in CO are paraffins, calendin, and calendulin [12]. All these secondary metabolites increase the importance of CO as traditional medicine. Carotenoids and triterpene alcohols, in both free and esterified forms, are also present in CO [24]. Co-derived carotenoid pigments [25] and other polyunsaturated fatty acids produced from CO, such as Calendric acid [26], have been demonstrated to have anti-inflammatory activities in vitro and in vivo [27]. Nonetheless, triterpene oligoglycosides and calendasaponins A, B, C, and D from CO have proven to exhibit gastric-emptying-inhibitory, gastroprotective, and hypoglycemic properties [28]. CO leaf extract contains fatty acids, triterpenes, chloroform extracts, and sterols. In the aqueous extract, flavonoids and saponins were identified, and alkaloids were also found in the ethanolic extract. In various regions of CO, the compounds ubiquinone, tocopherol, phylloquinone, and proto-quinone were identified from quinones. From the petroleum ether extract of CO flowers, terpenoids were extracted [22,23]. CO was also used to extract flavonoids such as quercetin, isorhamnetin, and isoquercetin. Some other phytoconstituents present in CO are paraffins, calendin, and calendulin.

Table 1 represents the major constituents and percentages of various *Calendula* species, and Table 2 represents the various chemical constituents present in CO.

### 2.1. Carotenoids 

The flower of CO, which is primarily orange, has high levels of carotenoids. The number of carotenoids in CO inflorescences increased significantly. Orange CO species include more hydrocarbons than yellow ones, which mainly contain oxygenated derivatives [25].

Carotenoids, which are pre-eminently found in plant flowers, majorly consist of lycopene, beta carotene, lutein, flavoxanthin, and zeaxanthin. Some of the other carotenoids found in petals and pollens of CO are luteoxanthin, neoxanthin, violaxanthin, 9Z-Violaxanthin, 9Z-Neoxanthin, auroxanthin, 9Z-Anthroxanthin, mutatoxanthin, 13/13′Z-Lutein, α-cryptoxanthin, z-cryptoxanthin, 9/9′Z-lutein, α-carotene, *β*-carotene, and *β*-cryptoxanthin. In addition to this, carotenoids found in the stem and leaves of CO include violaxanthin, 9Z-Violaxanthin, 9Z-Neoxanthin, antheraxanthin, neoxanthin, mutatoxanthin epimer 1 and 2, 9/9′Z-Lutein, *β*-carotene, α-cryptoxanthin, lutein, luteoxanthin, *β*-cryptoxanthin, and 13Z-Violaxanthin [51,52]. Carotenoids are predominantly known for their antioxidant activity through a radical scavenging mechanism, which makes them extremely useful in the pharmacotherapy of oxidative disorders. The same antioxidant potential and their ability to form artificial cross-linkage make them possess wound-healing action [25,53,54]. Zeaxanthin, a non-provitamin A carotenoid belonging to the xanthophyll family, is known to have a beneficiary therapeutic effect on age-related macular degeneration through its antioxidant and blue-filtering potential [55].

### 2.2. Terpenoids

Terpenoids, which are primarily present in flowers and roots of CO, are majorly known for their antioxidant activity. These sesquiterpenoids, entailing τ-cadinol, α-cadinol, and τ-muurolol, bring out antioxidant action through a radical scavenging mechanism. Thus, terpenoids have a significant role in the management of diseases and disorders involving oxidative reactions such as Alzheimer’s disease, skin hyperpigmentation, and diabetes-related complications. In addition to this, terpenoids have extensive anti-inflammatory action. This action is brought about by the inhibition of the COX-2 enzyme (Cyclo-oxygenase-2), pro-inflammatory cytokines including Interleukins 1 and 6, tissue necrosis factor, and synthesis of prostaglandins [22,23].

### 2.3. Flavonoids

Flavonoids present in CO, especially quercetin, have significant wound-healing activity. There are several proposed mechanisms for this action. The basic mechanism of action is the antioxidant activity brought about through radical scavenging action. Another mechanism explains the adhesion and augmentation of fibroblasts, which additionally cause an approbatory effect on the cellular activity in a given area [56,57]. Additionally, flavonoids are known to have anti-plaque and anti-gingivitis action [58,59]. Quite a few mechanisms are suggested for this action, including removal of plaque through inhibition of lysosomal hydrolase, reduction in collagen degradation through inhibition of recombinant human matrix metalloproteinases (MMPs), and subsequent increment in collagen concentration. Furthermore, other constituents of CO belonging to the flavonoid class such as rutin, apigenin, kaempferol, vitexin, and luteolin are known to have skin-protective action through the antioxidant mechanism. In addition to this, flavonoid compounds quercetin and rutin possess anti-depressant action. These compounds produce their action through the inhibition of Monoamine (MAO) oxidases and reduction in GABA levels [60,61,62]. Isorhamnetin with quercetin derivatives such as 3-*O*-(2″-rhamnosyl)-rhamnosides, 3-*O*-(2″-rhamnosyl)-glucosides, 3-*O*-(2″,6″-di-rhamnosyl)-glucosides, 3-*O*-(6″-rhamnosyl)-glucosides, 3-*O*-glucosides, and 3-*O*-(6″-acetyl)-glucosides are known to have anti-acetylcholinesterase activity. This activity is chemically attributed to the presence of acetyl and rhamnosyl groups in flavonoid structure [27]. Hyperoside, another flavonoid class compound, has a crucial effect on the management of osteosarcoma through restraining multiplication and stimulation of osteogenic differentiation of sarcoma cells [63].

### 2.4. Coumarins

Coumarins, which are significantly found in flowers of CO, may prevent oxidative damage to cells [54]. Some of the important coumarins present in CO are Umbelliferone, Scopoletin, and Esculetin. Umbelliferone, using the antioxidant mechanism of action, acts as a skin-protective agent, especially in sunscreen products. Scopoletin has spasmolytic action: it acts by constraining the spastic contraction of muscles of the urogenital system and gastrointestinal system. Esculetin, which is from the same class of coumarins, acts as a phlebotonic and inflammatory agent by decreasing the permeability of capillaries and rejuvenating venous tone. In addition to this, it is also known to have anti-thrombotic action owing to its revelatory effect in the augmentation of the occlusion period for thrombotic platelet plug formation [64,65].

### 2.5. Phenolic Acids

Phenolic acids found in CO such as caffeic acid, vanillic acid, chlorogenic acid, and coumaric acid have proven scavenging activity because of their hydrogen-donating tendency, which makes them useful in the treatment of oxidative disorders [66]. The same mechanism of action explains the role of phenolic compounds from CO in the reduction in exercise-instigated oxidative stress [67,68,69]. CO contains various fatty acids such as calendic acid, linoleic acid, oleic acid, palmitic acids, and dimorphecolic acid. Among all fatty acids with a crucial role in human bodily functions, calendic acid, a major fatty acid found in CO, has additional predominant cytotoxic action. The mechanism of this action is lipid peroxidation and downregulation of the gene lcf1, which is involved in the encoding of long-chain fatty acyl-CoA synthetase [70,71,72].

Some of the fatty acid derivatives from CO are 28-*O*-*β*-*D*-glucopyranosyl ole-anolic acid 3-*O*-*β*-*D*-galactopyranosyl (1→3)-*β*-*D*-glucuronopyranoside (calendulaglycoside C) [73], 28-*O*-*β*-*D*-glucopyranosyl oleanolic acid 3-*O*-*β*-*D*-glucuronopyranoside (chikusetsusaponin or glycoside F) [74], oleanolic acid 3-*O*-*β*-*D*-galactopyranosyl (1→3)-*β*-*D*-glucuronopyranoside (calenduloside G) [75], 28-*O*-*β*-*D*-glucopyranosyl oleanolic acid 3-*O*-*β*-*D*-glucopyranosyl-(1→2)-[*β*-*D*-galactopyranosyl-(1→3)]-*β*-*D*-glucuronopyranoside (calendulaglycoside A) [42], 28-*O*-*β*-*D*-glucopyranosyl oleanolic acid 3-*O*-*β*-*D*-galactopyranosyl (1→3)-*β*-*D*-glucopyranoside (calenduloside B) [73], oleanolic acid 3-*O*-*β*-*D*-glucopyranoside (Glucoside I) [76], 3-*O*-*β*-*D*-glucopyranosyl-(1→2)-[*β*-*D*-galactopyranosyl-(1→3)]-*β*-*D*-glucopyranosyl olea-nolic acid (osteosaponin-I) [77], oleanolic acid, 3-*O*-*β*-*D*-galacto-pyranosyl-(1→3)-*β*-*D*-glucopyranosyl oleanolic acid (arvensoside B) [78], stigmasterol and machaerinic acid 3-*O*-*β*-*D*-glucuronopyranoside [79].

### 2.6. Quinones

Quinones that are majorly found in the leaves of CO consist of phylloquinone, α-tocopherol, ubiquinone, and plastoquinone. They have anti-cancer potential, and their mechanism of action is alkylation and cleavage of DNA through DNA topoisomerase I and II [80,81].

Other minor components of CO such as phenolics and tannins are known to have antioxidant action as well as anti-ulcer action, which is rendered through maintenance as well as regeneration of gastric mucosa [82].

### 2.7. Amino Acids

Threonine, glutamic asparagine, leucine, proline, acid, serine, histidine, phenylalanine, tyrosine, arginine, lysine, aspartic alanine, methionine, and valine are some of the amino acids that are present in CO that have been detected in its stems, leaves, and flowers. Around 5% of amino acids were found to be present in the leaves, 3.5% in the stems, and 4.5% in the flowers [83].

**Figure 1:**
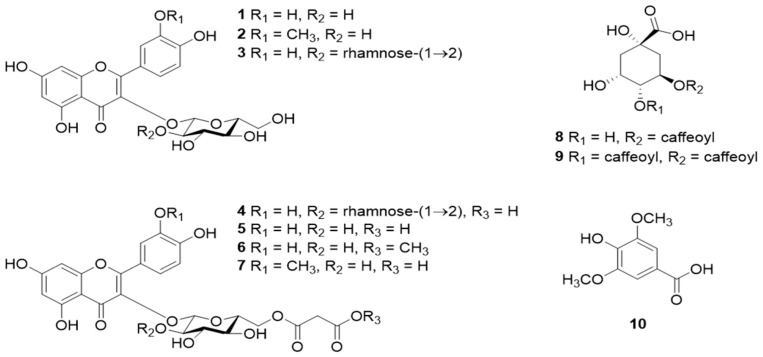
Chemical structures of constituents of CO. (**1**), isorhamnetin 3-*O*-*β*-glucoside (**2**), quercetin 3-*O*-*β*-neohesperidoside (**3**), quercetin 3-*O*-(2″-*O*-*α*-rhamnosyl-6″-*O*-malonyl)-*β*-glucoside (**4**), quercetin 3-*O*-(6″-*O*-malonyl)-*β*-glucoside (**5**), quercetin 3-*O*-6″-*O*-methyl malonyl)-*β*-glucoside (**6**), isorhamnetin 3-*O*-(6″-*O*-malonyl)-*β*-glucoside (**7**), chlorogenic acid (**8**), 3,4-dicaffeoylquinic acid (**9**), and syringic acid (**10**). Adapted from [84] under Creative Commons CC BY license (CC BY 4.0).

The chemical structures of some of the important chemical constituents present in CO are shown in Figure 1.

For the extraction of CO, numerous conventional and novel techniques are available. Conventional techniques used in past decades are hydrodistillation, solvent extraction, steam distillation, acid-catalyzed extraction, maceration, expression, and soxhlet extraction [85,86,87,88,89]. The most common solvents used in these processes are methanol, ethanol, acetone, and hexane. Here, due to the presence of high phenolic content, solvents with high polarity are employed; for example, 80% methanol, when used in the extraction process, leads to higher output in terms of the components yield [15,23,90]. Nevertheless, these conventional techniques are challenging because of prolonged extraction time, temperature, and pressure conditions. Here novel techniques such as ultrasound-assisted extraction, microwave-assisted hydrodistillation, microwave distillation, headspace solid-phase microextraction, and headspace–cold finger extraction come into the picture, which support numerous aspects such as enhanced extraction, in terms of yield and quality of extracts, and economical as well as ecological advantages [86,91,92,93,94].

## 3. Therapeutic Applications of *Calendula officinalis*

**Figure 2:**
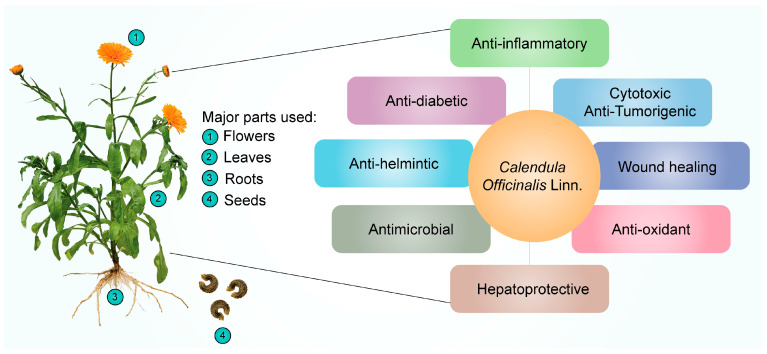
Pharmacological effects of *Calendula officinalis* Linn.

Many ailments have been treated with CO; a plant frequently used in homeopathic medicine. Additionally, it can be cytotoxic and inhibit tumor growth [95]. It functions as an antimicrobial [56,96], antioxidant [97], anti-inflammatory [89,98], antiseptic [99], anti-viral [89], hepatoprotective [56], and antidiabetic medicine [100]. It is also applied to the skin to treat various conditions, including inflammation of the skin, open wounds, and laceration wounds that bleed. Additionally, it is used to heal minor ailments such as razor burns and wind burns. The major parts of the CO plant and their therapeutic applications discussed in this review are represented in Figure 2 and Table 3.

### 3.1. Anti-Inflammatory

CO is currently being investigated, as it exhibits excellent anti-inflammatory activity. Alkaloids, tannins, flavonoids, essential oils, sterols, saponins, carotenoids, triterpene alcohols, mucilage, polysaccharides, and resin are only a few of the categories of secondary metabolites that the plant has that are correlated with the anti-inflammatory characteristics [110]. Dried flower heads or dried ligulate flowers are plant components that are utilized in medicine and cosmetics. The ligulate flowers are rich in triterpene alcohols, triterpene saponins, fatty acid esters, flavonoids, carotenoids, coumarins, hydrocarbons, essential oils, and fatty acids [111]. Using in vivo pharmacological testing, it has been determined that the triterpenoid fatty acid esters are responsible for the anti-inflammatory effects of *Calendula* flowers. The lauryl, myristoyl, and palmitoyl esters of faradiol are the most prevalent of these [112], demonstrating that flower extract of CO was much more effective for treating both acute (caused by dextran and carrageenan) and chronic (caused by formalin) swelling in mice. They hypothesized that it may be attributed to the inhibition of the production of proinflammatory cytokines (IL-6, interleukin 6; IL-1β; TNF-α, tumor necrosis factor α; and IFN-γ, interferon γ) and COX-2 (cyclooxygenase 2), and subsequently, Refs. [112,113] demonstrated the anti-inflammatory activity of CO extract and investigated its effects on nitric oxide production. The results revealed that the CO extract inhibited nitric oxide production in a dose-dependent manner, with cytotoxicity only observed at 147 μL/mL concentrations or above. 

**Figure 3:**
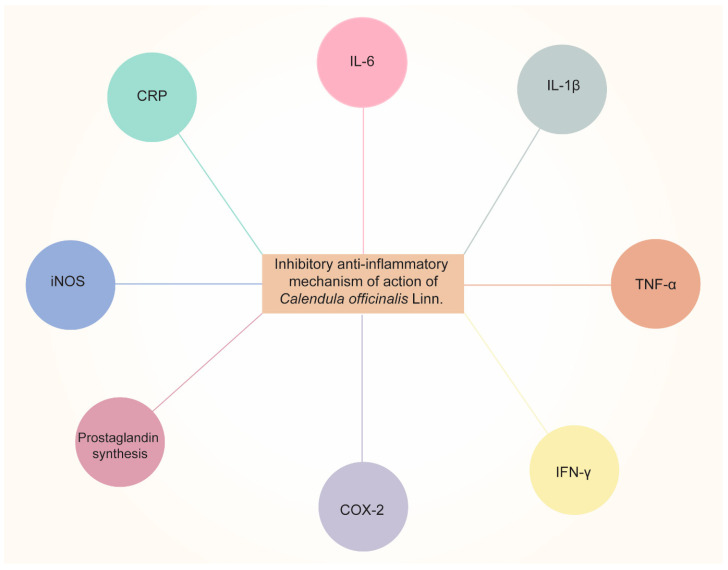
Anti-inflammatory effects of *Calendula officinalis* Linn by inhibiting pro-inflammatory cytokines (IL-6, IL-1β, TNF-α, and IFN-γ, etc.), COX-2, prostaglandin synthesis, iNOS (inducible nitric oxide synthase), and CRP (C-Reactive Protein).

Garrido-Suárez [98] studied the antinociceptive effects of CO cream on inflammatory hyper-nociception. Rats were subjected to several tests, and it was reported that CO cream (20% or 30% *w*/*w*), when applied topically, led to a significant decrease in TNF-α and suppression of COX-2. Pharmaceutical formulations such as nanoemulsion [114] have also been developed to achieve the anti-inflammatory effects of CO. Furthermore, the scientists discovered that all three samples of *Calendula* extract (3, 5, and 7%) had beneficial effects on healing and soothing wounds when applied to albino rats. The *Calendula* extract nanoemulsion has an anti-inflammatory impact on skin cells, according to the findings. The schematic representation of the anti-inflammatory effects of CO is shown in Figure 3. The aforementioned information reveals the potential uses of CO as an anti-inflammatory and analgesic agent. Considering this characteristic of CO, it was able to minimize dermatitis in newborns caused by diaper friction when compared to Aloe vera [101]. In the oral cavity, mouth rinsing with CO tincture reduced gingival inflammation [58]. 

### 3.2. Antioxidant Activity

Plant polyphenols such as flavonoids are among the most significant natural compounds with active antioxidant properties. The radical scavenging or chelating flavonoids are caused by their hydroxyl group content [115,116]. The family of antioxidants [115] as phenolic chemicals, on the other hand, operate as free radical terminators [117]. Hence, CO’s high flavonoid and phenolic phytochemical content contribute to its antioxidant activity, which can further promote its strong radical-scavenging capacity and confer protective effects [104]. The leaves and petals of the CO plant contain natural sources of antioxidants [56]. As a result of riboflavin’s photoreduction, it has been claimed that CO extract scavenges hydroxyl and superoxide radicals. Pandey et al. [118] examined the antioxidant properties of the leaves and flowers of CO by using TBA (thiobarbituric acid) and FTC (ferric thiocyanate) techniques. The FTC technique calculated the amount of peroxide produced during the initial stage of linoleic acid peroxidation. The results revealed that the antioxidant concentration decreases with decreasing absorbance value. When compared to regular Vitamins C and E, the aqueous extract of leaves and petals exhibited a high level of antioxidant effect based on absorption rates. The fact that the aqueous extract of the petals displayed lower absorbance with both the FTC and TBA techniques suggests that the petals possessed more antioxidant activity than the leaves.

Based on the evidence, it can be concluded that CO extracts may be extremely beneficial in treating several ailments such as AIDS (acquired immunodeficiency syndrome), heart disease, malaria, diabetes, stroke, cancer, and arteriosclerosis due to their potent antioxidant activity.

### 3.3. Cytotoxic and Anti-Tumor Activity

Saponin, one of the separated active compounds of CO, has been shown to exhibit antimutagenic action [119]. The interest in the purported anti-tumor activity of CO extracts and components has grown with the rise of complementary and alternative medicine based on herbs as cancer treatment. Cruceriu et al. [120] demonstrated the anti-tumor activity of methanolic extracts of CO using a cell line study. The authors reported that CO extracts could exert anti-cancer activity by inducing apoptosis, activating caspase 3 and caspase 7 at a protein level, and downregulating cyclin D1, D3, A, E, and several cyclin-dependent kinases. Furthermore, BAX (Bcl2 associated X protein) and BBC3 (Bcl2 binding component), two proapoptotic genes, were upregulated and NF-κB (nuclear factor kappa-light-chain enhancer of activated B cells) and STAT3 (signal transducer and activator of transcription factor 3) were downregulated after the treatment with CO extracts. Similarly, Hernández-Rosas et al. [121] demonstrated the in vitro cytotoxic effects of hydro-alcoholic extract of CO on human cancer cell lines. The authors found that the biological activities of high free-radical scavenging capacity (ABTS; 2,2-azino-bis (3-ethylbenzothiazoline-6sulfonic acid, DPPH; 2,2-diphenylpicrylhydrazyl), moderate ability to neutralize hydroxyl radicals, effective metal chelation, and strong reducing capacity are responsible for the anti-cancer effect. 

Clinical studies have shown the use of CO in different presentations. At the beginning of the 20th century, the clinical study conducted by Pommier et al. [102] showed the efficacy of *Calendula* ointment as adjuvant therapy when compared to trolamine for acute dermatitis during irradiation in the treatment of breast cancer. In another study, promising results showed the use of CO gel on oral leukoplakia when compared to lycopene gel [103]. In oral mucositis, the 2% CO mouthwash was able to decrease oral mucositis when compared to the placebo group [104].

In conclusion, there are encouraging findings about CO’s prospective usage in cancer management, particularly in cancer prevention, treatment of cancer, and palliative care for cancer patients. However, progress to pertinent preclinical studies is impeded without understanding the bioactive components responsible for the in vitro and in vivo selective cytotoxicity and for preventing radiotherapy-induced adverse effects. As a result, further study is required to find novel components of CO that have the potential to become useful bioactive components in the treatment of cancer.

### 3.4. Wound-Healing Activity 

Chronic wounds and delayed wound healing are major medical issues that provide difficult clinical challenges for doctors and have profound socioeconomic consequences. Since ancient times, herbs and their preparations have been utilized in addition to traditional medicines to expedite the healing of wounds. In this context, preparations (alcoholic and lipophilic) made from the flowers of CO have received stellar reviews for treating mild skin inflammations and slow-healing wounds. This is accomplished by enhancing the amount of blood and oxygen delivered to the wound site, which encourages the body to produce new tissue. CO plants’ dried petals are used to make tinctures, ointments, and washes to cure mild infections, scrapes, bruises, and burns. CO also contributes to maintaining calmed, hydrated skin by encouraging the development of collagen, a necessary protein for radiant skin. 

**Figure 4:**
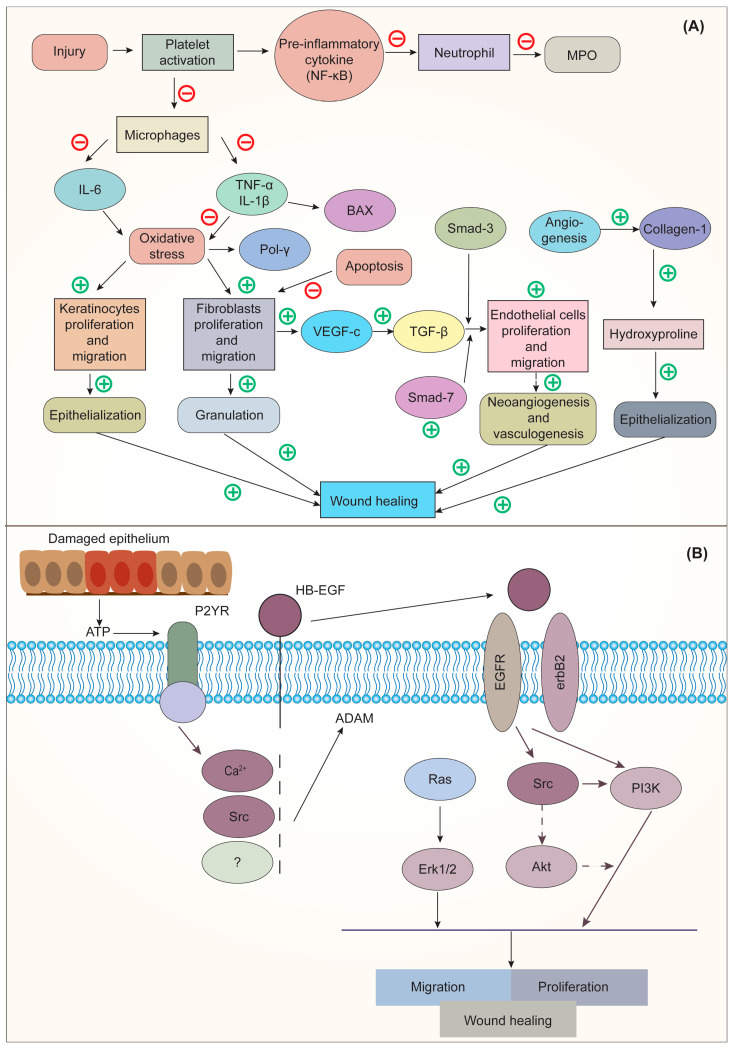
(**A**). Mechanism of action of CO on Interleukin 6 (IL-6); (**B**) Mechanism of action of epidermal growth factor (EGF) on wound healing. Adapted from [122] under Creative Commons CC BY license (CC BY 4.0). NF-κB, nuclear factor kappa-light-chain-enhancer of activated B cells; MPO, myeloperoxidase; IL-6, interleukin-6; TNF-α, tumor necrosis factor alpha; IL-1β, interleukin-1-beta; BAX, BCL-2 associated x protein; Pol γ, DNA polymerase γ; SMAD, suppressor of mothers against decapentaplegic; VEGF-c, vascular endothelial growth factor C; TGF-β, transforming growth factor-beta; ATP, adenosine triphosphate; P2YR, purinergic G protein-coupled receptors; HB-EGF, heparin-binding EGF-like growth factor; EGFR, epidermal growth factor receptor; RAS, rat sarcoma; ERK1/2, extracellular signal-regulated kinase; Src, steroid receptor coactivator; Akt, protein kinase B; PI3K, phosphoinositide 3-kinase.

Deka et al. [99] stated that CO could dramatically increase wound angiogenesis and collagen metabolism, which results in scar softening and emollient characteristics. The floral extract of CO, when applied topically and orally, has therapeutic properties for burns and wounds. An increase in collagen-hydroxyproline and hexosamine shows that the person or animal being treated is mending their wounds. Gunasekaran et al. [122] demonstrated the wound-healing activity of CO in the winter strain of albino rats. The results revealed that a herbal ointment containing CO could inhibit the activation of macrophages and speed up the migration and proliferation of keratinocytes and fibroblasts, which were responsible for wound healing. This was accomplished by preventing the release of proinflammatory cytokines and reducing oxidative stress at the wound site. The mechanism of action of CO for wound healing is shown in Figure 4.

Similarly, Rathod and co-workers [123] investigated the wound-healing efficacy of CO-loaded collagen films on wounds induced in Wistar rats. On day 21, the rate of wound contraction in the developed CO film was considerably higher than in the control group, the placebo-treated group, and the marketed-product-treated group. In a randomized controlled trial, the CO-containing ointment was studied on 72 qualified primiparous females for cesarean wound healing. According to the findings, applying CO ointment to the wound after a cesarean significantly boosted the rate of wound healing. It can be successfully employed to speed up the cesarean healing process [124].

It is important to note that clinical studies have already been conducted in order to evaluate the efficacy of the use of CO in the healing of hand and finger wounds by secondary intention. In this perspective, there is evidence showing that CO extract is favorable for the treatment of these wounds by reducing the epithelialization time and increasing the healing speed [105]. In chronic wounds, such as venous ulcers, the use of CO also obtained positive results, showing that the treatment with topical CO reduces the surface area of the lesion, achieves greater epithelialization in less time, and accelerates healing time [106]. In addition, this type of healing is advantageous because it reduces medical interventions and treatment costs [125]. Another important finding is that the use of CO ointment after episiotomy reduces pain, redness, and swelling and helps healing [107].

### 3.5. Hepatoprotective Activity

Most substances that enter the body are processed by the liver, which is also in charge of detoxification. Up to 83% of all pathological cases worldwide are hepatotoxic, making it the most prevalent disease. The main causes of liver toxicity include hepatitis, viral infections, dietary additives, alcohol, toxic industrial chemicals, air pollution, and water pollution. Researchers have shown that CO extracts can protect the liver from the cytotoxicity and oxidative stress caused by carbon tetrachloride. This results in a rise in the amount of total hemoglobin. Similarly, in vitro and in vivo models of the flowers’ hydro-alcoholic extract show decreased hepato-cytolysis and liver biomarkers. The treatment with ethanolic extract brought back normal levels of hepatic blood markers, increased the level of total thiols, decreased levels of total antioxidant status, decreased levels of antioxidant enzymes (CAT, catalase; SOD, superoxide dismutase; GPx, glutathione peroxidase; and GST, glutathione s-transferases) and decreased the levels of malondialdehyde and total oxidant status in both the blood and the hepatocytes. Furthermore, restoration of cellular antioxidant levels, specifically enhanced levels of reduced glutathione enzymatic components and total thiols of the antioxidant system, was also observed, which may be due to the polyphenolic chemicals in CO that protect the cells from chemically induced cellular damage. Moreover, in a dose-dependent manner, CO extract improved the histological picture of the liver, as well as the biochemical parameters and inflammatory cytokines [126]. 

### 3.6. Anthelmintic Activity

In addition to being a major cause of illness in humans and animals, parasitic infections also negatively impact the economy. Due to increased resistance to conventional antihelminthic treatments, there has been a quantum leap toward investigating herbal medicines. Herbs such as CO have been used for centuries to combat parasitic illnesses, and they are still utilized for that purpose in many countries. In a study, Khursheed et al. [89] investigated the anthelmintic activity in adult Indian earthworms (*Pheretima posthuma*). It was observed that the ethanolic extracts of CO exhibited anthelmintic activity (paralysis of the worms followed by death) at 10 mg/mL concentration compared with the standard drug, albendazole. CO was also proven to show anthelminthic activity against *Ascaris suum* [127] and 50% efficacy on L1-2 larvae of *Strongiloides papillosus* [128].

### 3.7. Antimicrobial Activity

Although antibiotics have played a significant part in the treatment of infectious diseases caused by bacteria and fungi for the past 60 years, it has been observed that the occurrence of dangerous bacteria that are resistant to antibiotics has increased in frequency over the course of the past several decades [129]. Because there are a number of different mechanisms by which drug resistance can be manifested, finding a solution to this issue is not likely to be an easy challenge. Because of the growing prevalence of drug-resistant pathogens, there is an immediate and pressing requirement to discover and isolate new bioactive compounds derived from medicinal plants using standardized and contemporary analytical methods. Compounds obtained from medicinal plants might provide unique and relatively simple techniques to treat pathogenic microbes. CO extracts have also proven to be effective as antimicrobial agents [36].

#### 3.7.1. Antibacterial Activity

It is of the utmost significance to discover novel antibacterial medicines in view of the research that shows the rapid global spread of clinical isolates that are resistant to antibiotic treatment. A large variety of medicinal plants have been identified as useful sources of natural antibacterial agents as alternative choices that have the potential to be successful in the treatment of several bacterial diseases [130]. The antibacterial properties of a variety of plants, which are caused by the production of phytochemicals during the secondary metabolism of the plant, have led to their adoption in a wide range of fields. Tannins, alkaloids, phenolic compounds, and flavonoids are just examples of the large range of secondary metabolites that are abundant in plants. These metabolites have been shown to exhibit antibacterial effects when tested in vitro [131,132,133]. 

CO has also been shown to possess potent antibacterial properties. Recently, Karnwal [134] studied the antibacterial potential of CO. It was observed that the minimum inhibitory concentration (MIC) with CO aqueous extracts was 3.75 % for *Clostridium perfringens*, *Staphylococcus aureus*, *Pseudomonas aeruginosa*, and *Listeria monocytogenes*. For *Listeria monocytogenes*, *Clostridium perfringens*, and *Staphylococcus aureus*, however, the lowest MBC (1.87%) was observed. Ethanolic extract of CO showed the lowest MIC and MBC for just one bacterial pathogen, and that was *Pseudomonas aeruginosa*, with 3.75% and 1.87%, respectively. The antibacterial property of CO was also compared to sodium hypochlorite against *Streptococcus mutans* as a root-canal-irrigating solution by Yalgi et al. [135]. It was observed that CO showed a significant CFU reduction in *S. mutans*, i.e., from 15.85 CFU to 1.20 CFU. The results were comparable to those of the group treated with sodium hypochlorite. The authors reported that the antibacterial effect may be attributed to the presence of terpene alcohols and terpene lactones in CO. Darekar et al. [136] also investigated the antibacterial potential of CO against *Bacillus subtilis*, *Klebsiella pneumonia*, *Staphylococcus aureus*, and *Enterococcus faecalis* using the disc diffusion method at a concentration of 10 mg/mL. The results revealed strong antibacterial activity of CO against the tested strains as indicated by their significant inhibition zones. Shahen and coworkers [137] studied chemical compounds with bioactive properties from CO flowers and their antibacterial activity. The authors studied paper-disc agar diffusion and tube-dilution techniques to test growth inhibition and to calculate the minimum inhibitory concentration. Variable levels of antibacterial activity were shown by the leaf extract against various microorganisms. The largest inhibitory zone was generated by *E. coli* and *K. pneumonia* around the CO leaves, whereas *B. subtilis* and *S. lutea* were shown to be more resistant bacteria. *E. coli* had the least inhibitory effects. Calendula extracts in petroleum ether and chloroform showed antibacterial efficacy against *B. subtilis* and *E. coli*. This finding shows that several pathogens are strongly inhibited by leaf extracts of CO that were made using petroleum ether and chloroform.

#### 3.7.2. Antiprotozoal Activity

An important global cause of death and morbidity is protozoal disease. Every year, malaria infects between 200 and 500 million people, killing 2 million of them, mostly young children under the age of 5 [138]. Future therapeutic agents must be found immediately, and understanding traditional medicine can help to pave the path for future developments in this area. However, due to the restricted availability and high cost of pharmaceutical treatments, it is estimated that two-thirds of the global population relies on traditional medicines. Additionally, it was discovered through global biological screens that many natural compounds had antiparasitic activities, often with a surprising potency and high selectivity. Samra et al. [139] studied the antiprotozoal activity of CO. The methanol extract of CO included a novel phytoconstituent called (6Z,9Z)-heptadeca-6,9-diene-5,11-dione (I). The structure of I was discovered by examining NMR spectra and HRESIMS data. For both antibacterial and antiprotozoal properties, tests were conducted. Compound I demonstrated mild antitrypanosomal activity with an IC50 of 37.6136 µM, leishmanicidal activity against *L. donovani* amastigote with an IC50 of 16.4394 µM and IC90 of 28.9015 µM, and leishmanicidal activity against *L. donovani* ecdysone. Standard experimental techniques were used to test compound I cytotoxicity against THP1 cells; however, no cytotoxicity was seen, demonstrating its selectivity and safety. 

#### 3.7.3. Antifungal Activity

According to the findings of the epidemiological studies, the incidence and prevalence of major fungal infections are likely to continue to be a concern for public health. Antifungal treatments have been used more often, which has resulted in the emergence of fungal strains that are resistant to these medications. It is vital to identify new classes of antifungals from natural products such as medicinal plants because of the rapid growth of drug-resistant strains of fungus that are resistant to several treatments. CO has been found to possess antifungal properties [140]. Vinola et al. [97] compared the antifungal activity of CO with 2% chlorhexidine against *C. albicans*. It was observed that compared to CO, chlorhexidine exhibits much higher antifungal activity against *C. albicans*. CO does, however, have some antifungal efficacy against *C. albican*. Nevertheless, CO also displayed volume-dependent antifungal activity against *C. albican* to a considerable extent.

Recent findings shed light on the antifungal characteristics of CO that are utilized in the treatment of infectious disorders. The bioactive chemicals from CO extracts will need to be identified in further detail, as well as their pharmacological target or mechanism of action. It is now essential to conduct more clinical trials to thoroughly investigate the antimicrobial principles of CO and their numerous potential uses. It also has the potential to be employed for the preservation of processed foods. Various other therapeutic applications of CO are represented in Table 4.

## 4. Future Perspectives

For many centuries, CO has been utilized by humanity for diverse therapeutic applications. CO herb consist of terpenoids, steroids, phenolic compounds, carotenes, triterpenoids, essential oils, quinones, fatty acids, minerals, saponins, carbohydrates, and tocopherols, with α-cadinol (sesquiterpenoid) as a major component. Being rich in these secondary metabolites, CO has been proven to have anti-inflammatory, antidiabetic, antioxidant, anti-cancer, antibacterial, anti-ulcer, antifungal, anti-viral, anti-thrombogenic, neuroprotective, antiprotozoal, skin-protective, and antifatigue activities. Considering that CO has these multiple applications, it is crucial that extensive research on nonfloral components of the plant, such as seeds, roots, leaves, and stems, be conducted in the future. In addition to this, there is a vital need to focus on genus chemistry. Because of the limited amount of literature available, other species of CO including *C. arvensis*, *C. tripterocarpa*, *C. stellata*, and *C. suffruticose* should be explored further regarding their biochemical profiles and pharmacological properties. In the same fashion, relative studies should be conducted to understand variations in terms of the age of the plant, method of extraction, or processing method. It is anticipated that as extraction methods become more advanced, previously unidentified phytochemicals and an expansion of this plant’s pharmacological range of activity will likely be found, posing fascinating research challenges. Moreover, the research on developing novel drug delivery systems containing CO is still nascent; we anticipate that research in this area will continue. Molecular docking and molecular dynamics are two modern computational drug design techniques that hold great promise for developing novel therapeutic candidates for various ailments.

Additionally, bioinformatics technologies have opened up new avenues for finding the essential critical amino acids under almost comparable physiological settings, considerably validating the outcomes of computational methods. However, based on the chemical makeup of the medication and its target receptor, the therapeutic potential of several bioactive compounds can be investigated, saving time and money [150]. In the foreseeable future, CO-containing micro- and nano-formulations have excellent potential for treating several ailments, and the future developments and applications are assured to be astounding. Moreover, activity enhancement, combined with other available agents, offers a promising strategy that may ultimately enhance pharmacological outcomes [3].

## 5. Conclusions

CO species have shown tremendous health advantages from prehistoric times to the present. The present state-of-the-art CO in the health sciences realm has been rigorously examined and briefly explained in this study with insights into their molecular processes. Additionally, many CO-containing drug delivery methods and patents have been developed to improve solubility, targeting, and stability, and their active components have been considered in this analysis. As a result, it is envisioned that this review will act as a foundation for scientists, agronomists, and even small-scale herbal industries to integrate the information that is currently available on CO and realize the full pharmacological, agricultural, and industrial potential of this fascinating medicinal plant.

## Figures and Tables

**Table 1:** The major constituents and percentages of various *Calendula* species [29].

Species	Major Component	Percentage	References
*Calendula suffruticosa*	α-linolenic acid	24.20	[17,30]
*Calendula arvensis*	d-cadinene (Sesquiterpines)	15.1	[16,31]
*CO*	α-cadinol	64	[31,32,33,34,35,36]
*Calendula stellata*	linalool	34.40	[30]
*Calendula tripterocarpa*	Phenolic compounds	11.22	[37,38]

**Table 2:** Various chemical constituents are present in *Calendula* officinalis Linn.

Plant part	Groups	Active Ingredients	Ref.
Flower	Terpenoids	ψ-taraxasteol, Lupeol	[39]
		Erythrodiol	[40]
		Calenduloside	[41]
		*Calendula* glycoside A and B	[42]
		Cornulacic acid acetate	[43]
	Flavonoids	Calendoflavoside Isoquercitrin, rutin	[42]
		Isorhamnetin, Quercetin	[44]
		Narcissin, Isorhamnetin-3-*O*-*β*-*D* glycoside	[45]
	Coumarins	Scopoletin, umbelliferone, Esculetin	[46]
	Volatile oils	Oplopanone, Cubenol, methyl linoleate	[47]
		Limonene, nerolidol, palustron p-cymene, nonanal, Sabinene, carvacrol, α-pinene, t-muurolol, geraniol	[48]
Leaves	Quinones	α-tocopherol, plastoquinone, Phylloquinone, ubiquinone	[49]
Root	Terpenoid	Calenduloside B	[50]

**Table 3:** Summary of clinical studies of the use of *Calendula officinalis*.

Author and Year	Applicability	Outcomes	Reference
Panahi et al., 2012	Diaper dermatitis	This study suggests that topical use of CO could be used effectively for the treatment of diaper dermatitis in infants.	[101]
Khairnar et al., 2013	Dental plaque and gingival inflammation	The use of CO mouthwash was able to reduce dental plaque and gingivitis.	[58]
Pommier et al., 2004	Acute dermatitis	Topical use of CO prevented acute dermatitis grade 2 or higher in breast cancer patients given radiation therapy.	[102]
Singh and Bagewadi, 2017	Homogeneous leukoplakia	The use of CO extract gel was effective in reducing the size of the lesion.	[103]
Babaee et al., 2013	Oropharyngeal mucositis	The use of CO extract gel was able to reduce the intensity of oropharyngeal mucositis in patients undergoing radiotherapy during treatment for head and neck cancer.	[104]
Giostri et al., 2022	Acute wounds on hand	CO induced more rapid secondary intention healing in hand and finger wounds	[105]
Buzzi et al., 2016	Venous leg ulcer healing	Patients with ulcers treated with CO extract had a significant 4-fold increase in percentage healing velocity per week, compared with the control group.	[106]
De Angelis et al., 2022	Episiotomy	Women who used CO ointment after episiotomy had significantly lower pain level from the second day and during the entire follow-up. In addition, CO ointment also improves wound healing in terms of redness and edema.	[107]
Saffari et al., 2017	Vaginal candidiasis	Treatment of vaginal candidiasis with CO vaginal lotion seems to be successful.	[108]
Pazhohideh et al., 2018	Bacterial vaginosis	CO was used successfully and without any negative side effects to treat bacterial vaginosis in women of reproductive age.	[109]

**Table 4:** Various therapeutic applications of CO.

Therapeutic Application	Model	Results/Clinical Outcomes	Ref
Cardiovascular	Wister rats	In endothelium-depleted rat aortic rings pre-contracted with 60 mmol/L of KCl, the experiment revealed that floral extract of CO caused a concentration-dependent relaxation.	[141]
Hepatoprotective	Albino rats	The results showed that feeding on CO had a protective effect on the liver against CCl4 and had improvement effect on liver.	[29]
Antidiabetic	Albino wister rats	Significant changes occurred in glucose and insulin level (135.32 ± 2.43 and 88.42 ± 2.17, respectively) at a dose of 200 mg/kg/day in a dose-dependent manner as compared to the control positive group level (211.76 ± 3.95 and 134.82 ± 2.95) (*p* < 0.05).	[142]
Antidiabetic	Wister rats	In diabetic rats, the results revealed that CO was able to normalize levels of creatine kinase (CK-MB and total CK), amylase, and lipase. This allowed for a reduction in the negative effects of diabetes.	[143]
Polycystic ovary syndrome	Sprague–Dawley rats	After 21 days, the results demonstrated that rats given CO subcutaneous injections of DHEA were successfully induced with PCOS condition.	[144]
Wound healing	Swiss albino mice	In a wound model including excision, mice that had been treated with an extract of the leaves of CO showed a significant reduction in both the wound area and the time it took to epithelize.	[145]
Oral wound healing	Wister rats	The application of 10% CO gel improves wound contraction and enhances healing.	[146]
Wound healing	Wister rats	The healing percent of the lesion area ranged from 7.69% to 87.01% with CO-flower-extract-loaded hydrogel sheet.	[147]
Age-defying and photoprotective	Albino rats	According to the findings, a pre-treatment with GEO/CEO-encapsulated vesicular cream formulations significantly reversed the detrimental biochemical alterations and protected the skin from the deteriorating effects of UVB radiation.	[148]
Antifungal	Swiss albino rats	According to the findings, CO essential oils and their combination provide a significant advantage in terms of lowering the risk of fungal infection after chemotherapy with cyclophosphamide.	[149]

## Data Availability

Not applicable.
